# Relationships of ferroptosis-related genes with the pathogenesis in polycystic ovary syndrome

**DOI:** 10.3389/fmed.2023.1120693

**Published:** 2023-02-17

**Authors:** Shuang Lin, Xin Jin, He Gu, Fangfang Bi

**Affiliations:** Department of Obstetrics and Gynecology, Shengjing Hospital of China Medical University, Shenyang, Liaoning, China

**Keywords:** ferroptosis, PCOS, SVM-RFE, LASSO, bioinformatics

## Abstract

**Background:**

Numerous studies have suggested that ferroptosis plays a significant role in the development of polycystic ovary syndrome (PCOS), but the mechanism remains unclear.

**Methods:**

In this study, we explored the role of ferroptosis-related genes in the pathogenesis of PCOS using a comprehensive bioinformatics method. First, we downloaded several Gene Expression Omnibus (GEO) datasets and combined them into a meta-GEO dataset. Differential expression analysis was performed to screen for significant ferroptosis-related genes between the normal and PCOS samples. The least absolute shrinkage selection operator regression and support vector machine–recursive feature elimination were used to select the best signs to construct a PCOS diagnostic model. Receiver operating characteristic curve analysis and decision curve analysis were applied to test the performance of the model. Finally, a ceRNA network-related ferroptosis gene was constructed.

**Results:**

Five genes, namely, NOX1, ACVR1B, PHF21A, FTL, and GALNT14, were identified from 10 differentially expressed ferroptosis-related genes to construct a PCOS diagnostic model. Finally, a ceRNA network including 117 lncRNAs, 67 miRNAs, and five ferroptosis-related genes was constructed.

**Conclusion:**

Our study identified five ferroptosis-related genes that may be involved in the pathogenesis of PCOS, which may provide a novel perspective for the clinical diagnosis and treatment of PCOS.

## Background

Polycystic ovary syndrome (PCOS) is a complex disease characterized by reproductive, metabolic, and psychological features. It is usually diagnosed during the patients’ reproductive years and mainly presents with hirsutism, acne, irregular menstruation, and infertility (1). There are many theoretical hypotheses regarding the etiology of PCOS, and increasing evidence suggests that PCOS may be a complex polygenic disorder influenced by genetic factors, epigenetic variation, and the environment (2). The prevalence of PCOS ranges from 6 to 20%, depending on the populations studied and the definitions used (3, 4). To date, there is no uniform diagnostic standard or effective treatment for PCOS (5–7). Therefore, exploring the pathogenesis of PCOS can contribute to clinical diagnosis and therapies and improve reproductive outcomes. Numerous studies have shown that iron metabolism is related to endocrine diseases, including PCOS, but the underlying mechanisms remain unclear (8, 9).

Ferroptosis was first described in 2012 as a non-apoptotic, iron-dependent form of cell death, characterized by iron-dependent accumulation of lethal lipid reactive oxygen species (ROS) (10). Iron is essential for cellular biological processes, including growth, proliferation, and metabolism, among the many functions in the body. Balanced iron absorption, systemic transport, cellular uptake, and storage ensure balanced homeostasis of iron metabolism (11, 12). In recent years, ferroptosis is associated with the pathogenesis of various diseases, such as diabetes mellitus, renal failure, cardiomyopathy, neurodegeneration, ischemia–reperfusion injury, and cancer (13–18). Therefore, targeting ferroptosis has become a new research area for the design of therapies and disease prevention measures. Recent studies have shown that ferroptosis is involved in the pathogenesis of PCOS. Zhang et al. (5) reported that circRHBG inhibits ferroptosis in granulosa cell proliferation of PCOS through the circRHBG/miR-515/SLC7A11 axis. Liu et al. (19) revealed that the PCOS model *in vivo* and granulosa cells subjected to IR have increased ferroptosis levels and that the mechanism of cryptotanshinone in the treatment of PCOS is dependent on its inhibitory effect on cellular ferroptosis. Zhang et al. (20) indicated that ferroptosis proteins were associated with reproductive outcomes of POCS patients with infertility and constructed a FerSig risk prognostic model based on the expression of five independent prognostic ferroptosis proteins (G6PD, GPX4, PCBP1, DPP4, and PCBP2). Taken together, ferroptosis plays a key role in the development of PCOS; thus, comprehensive studies on ferroptosis genes in the pathogenesis of PCOS are urgently needed.

As of now, no studies have focused on the mechanism of ferroptosis-related genes in the pathogenesis of PCOS using comprehensive bioinformatics methods. Therefore, we identified differentially expressed ferroptosis-related genes (DEFRGs) in granulosa cells between normal and PCOS women using a public dataset downloaded from the Gene Expression Omnibus (GEO) database. Least absolute shrinkage and selection operator (LASSO) regression and support vector machine–recursive feature elimination (SVM-RFE) were used to select five hub DEFRGs to construct a PCOS diagnostic model, which was successfully verified in our clinical specimens. We then constructed a ceRNA network associated with the five hub DEFRGs. Our results may help illustrate the potential role of ferroptosis in the pathogenesis of PCOS and provide a novel perspective for the clinical diagnosis and treatment of PCOS.

## Materials and methods

### Data acquisition

**Figure 1:**
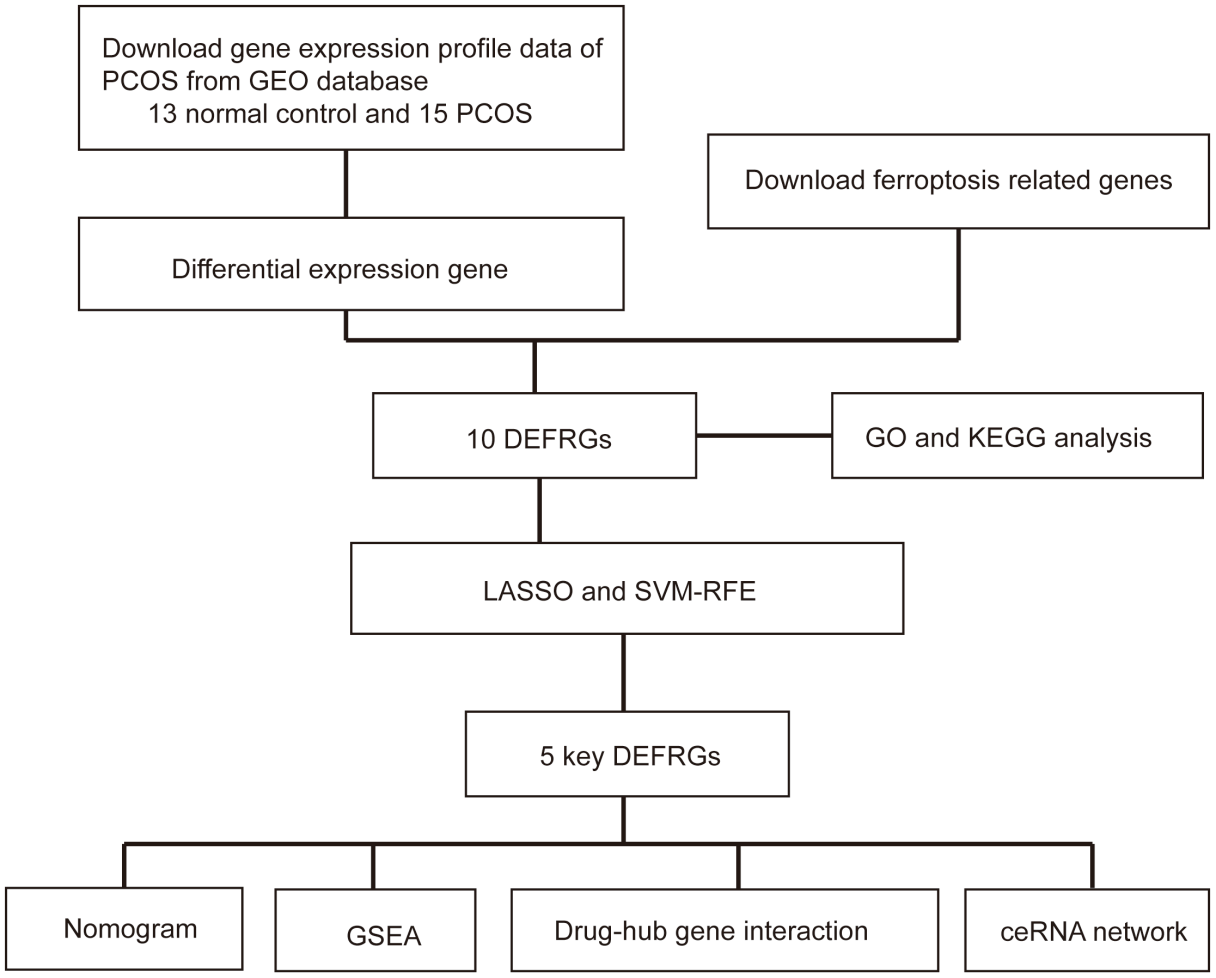
Flow chart of this study. PCOS: polycystic ovary syndrome; GEO: Gene Expression Omnibus; DEFRGs: differentially expressed ferroptosis-related genes; GO: Gene Ontology; KEGG; Kyoto Encyclopedia of Genes and Genomes; GSEA: gene set enrichment analysis; LASSO: least absolute shrinkage and selection operator; SVM-RFE: support vector machine–recursive feature elimination.

Based on the Affymetrix Human Genome U133A Plus 2.0 Array microarray platform, GSE5850, GSE34526, and GSE102293 gene expressions in granulosa cells from patients with PCOS were downloaded from the GEO database. The GSE5850 dataset consisted of six normal and six PCOS women. The GSE34526 dataset included three healthy controls and seven patients with PCOS. The GSE102293 dataset contained six samples, of which two were from patients with PCOS and four were from normal controls. We then combined and normalized the three datasets into a meta-GEO dataset using the “sva” R package and the “gcrma” R package. A flowchart of the study is presented in Figure 1.

### Differential expression analysis

We downloaded 820 ferroptosis-related genes (FRGs) from the FerrDb V2 website^1^. FRGs contained genes related to driver markers and suppressors. We identified differentially expressed genes (DEGs) between normal and PCOS women using the “limma” package in R. We then crossed the FRGs with DEGs to obtain DEFRGs for further investigation.

### Construction and evaluation of LASSO and SVM-RFE models

The least absolute shrinkage and selection operation and SVM-RFE algorithms were used to obtain key DEFRGs to diagnose PCOS based on the “glmnet” and “e1071” R packages (21). The LASSO algorithm was used to adjust the optimal value of the penalty parameter (*λ*) using a 10-fold cross-validation. The SVM-RFE algorithm determines the variable by searching for the lambda with the smallest classification error. Finally, key diagnostic genes for PCOS were identified by overlapping the diagnostic data from the two algorithms. Receiver operating characteristic (ROC) curves were drawn to demonstrate the diagnostic performance of the key DEFRGs, and the area under the ROC curve (AUC) was used to verify the efficiency and accuracy of the key diagnostic DEFRGs (7).

### Tissue collection

From January 2020 to December 2022, ovarian granulosa cells of 50 PCOS patients, 13 normal ovulatory women undergoing *in vitro* fertilization (IVF) at the ShengJing Hospital of China Medical University were collected. The diagnosis of PCOS met the Rotterdam 2003 diagnostic criteria: oligoovulation and/or anovulation, hyperandrogenism, and polycystic ovaries. Patients with congenital adrenal hyperplasia, Cushing’s syndrome, or androgen-secreting tumors were excluded. Control patients received IVF treatment for tubal disease, but had normal hormone levels, regular menstrual cycles, and normal ovarian morphology. COCs were isolated *via* ultrasound-guided vaginal puncture and washed in phosphate-buffered saline (PBS). Granulosa cells were selected from COCs. All the participants were under 40 years of age. This study was approved by the Ethics Committee of the ShengJing Hospital of CMU, and informed consent was obtained from all participants (No. 2020PS198K). The baseline information about all patients with PCOS is shown in Supplementary Table 1.

### Quantitative RT-PCR

Total RNA was isolated using TRIzol Reagent (TaKaRa, Shiga, Japan) and then reverse-transcribed into complementary deoxyribonucleic acid (cDNA) synthesis (PrimeScript™ RT Reagent Kit). Real-time qPCR was performed to detect gene expression using 2 × SYBR Green PCR Master Mix (Thermo Fisher Scientific, Waltham, MA, USA). The 2 − ΔΔCt method was used to calculate the relative gene expression. The sequences of the primers used for RT-qPCR are presented in Supplementary Table 2.

### Construction of a nomogram model

A nomogram model was conducted to facilitate the clinical application using the “rms” package. The “Points” indicate the score of each factor under different conditions, while the “Total Points” refer to the total score of all factors. Calibration curves were used to measure the predictive accuracy, and decision curve analysis (DCA) curves were used to evaluate the clinical value of the nomogram (22).

### Construction of the ceRNA network

To construct a lncRNA–miRNA–mRNA regulatory network based on the five key diagnostic DEFRGs, miRNA–mRNA and miRNA–lncRNAs interactions were predicted using miRWalk^2^ and miRDB^3^, respectively. The predicted miRNAs were intersected, and the lncRNA–miRNA–mRNA network was visualized using the Cytoscape software (version 3.8.2) (23).

### Functional enrichment analysis

To further explore the mechanism of DEFRGs in PCOS, Gene Ontology (GO) and Kyoto Encyclopedia of Genes and Genomes (KEGG) enrichment analysis were performed using the “clusterProfiler” package in R. Gene set enrichment analysis (GSEA) was conducted using c2.cp.kegg.symbols.gmt and c5.go.symbols.gmt (24).

### Statistical analysis

All statistical analyses were performed using the R software (version 4.0.2). We mapped the chromosomal positions of the key DEFRGs using the “RCircos” package in R and calculated the correlation coefficients between the key DEFRGs using the Spearman correlation analysis. The Wilcoxon rank-sum test was used to compare differences between the groups. Statistical significance was set at a two-tailed *p*-value of <0.05.

## Results

### Identification of differentially expressed ferroptosis-related genes in PCOS

**Figure 2:**
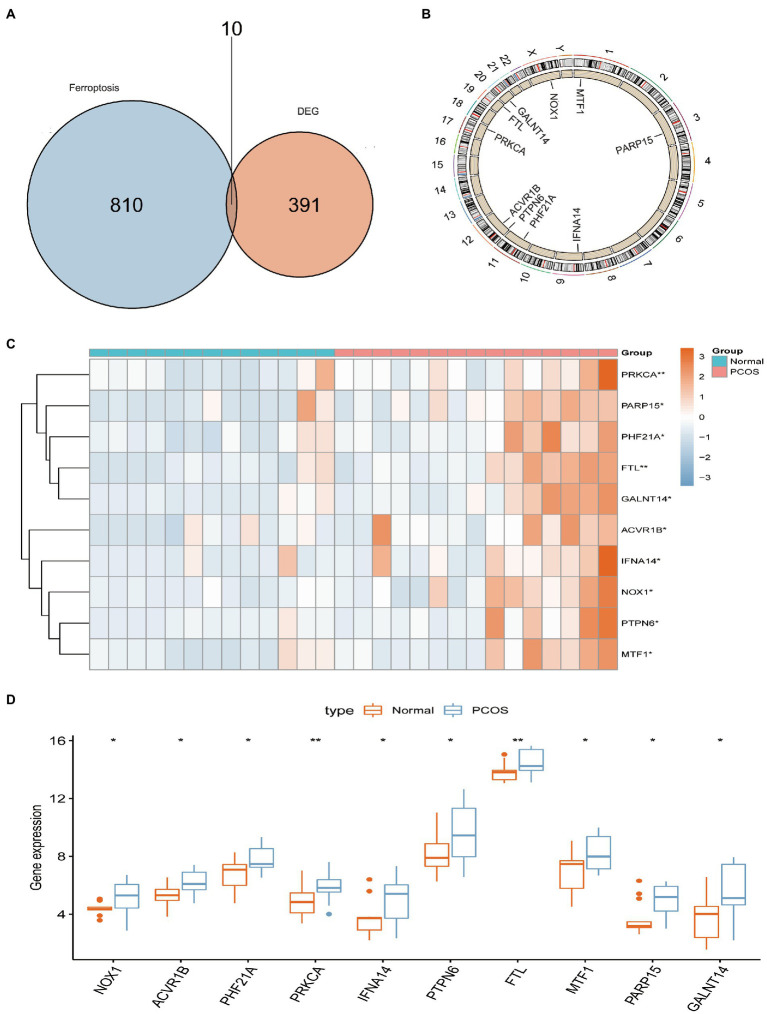
Landscape of 10 DEFRGs in PCOS. **(A)** Identification of 10 DEFRGs. **(B)** The chromosomal positions of the 10 DEFRGs. **(C)** Heat map of the expression of 10 DEFRGs between normal controls and patients with PCOS. **(D)** Histogram of the expression of 10 DEFRGs between normal controls and patients with PCOS.

**Figure 3:**
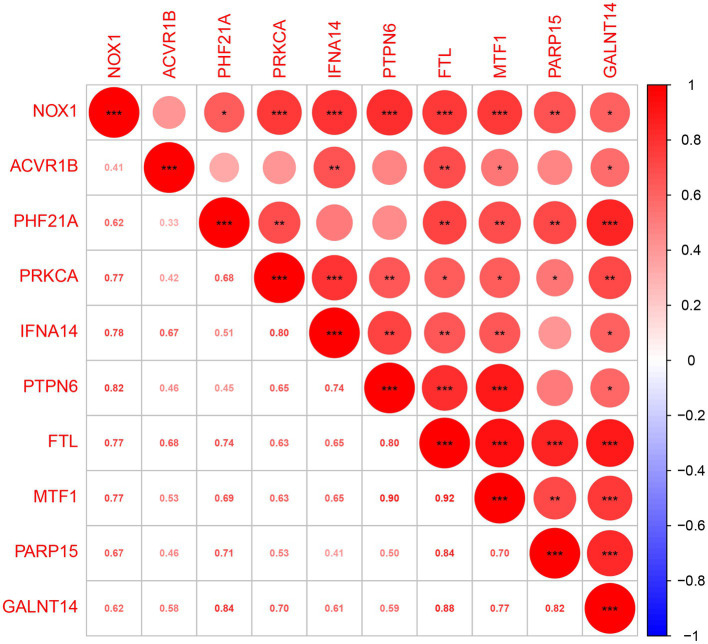
Spearman’s correlation analysis of 10 DEFRGs.

A total of 401 DEGs were identified between normal and PCOS women by the “limma” package according to the screening criteria of *p* < 0.05 and log |FC| > 1. We crossed 820 ferroptosis-related genes with 401 DEGs and obtained 10 differentially expressed ferroptosis-related genes (DEFRGs) (NOX1, ACVR1B, PHF21A, PRKCA, IFNA14, PTPN6, FTL, MTF1, PARP15, and GALNT14), which were visualized using a Venn diagram (Figure 2A). The circle diagram displays the chromosomal positions of the 10 DEFRGs (Figure 2B). Finally, the heat map and histogram were used to show the differential expression levels of the 10 DEFRGs between normal controls and patients with PCOS. We found that the expression levels of the 10 DEFRGs were upregulated in PCOS samples compared with those in normal controls (Figures 2C,D). The correlation heat map indicated that the 10 DEFRGs had a strong positive correlation with each other (Figure 3).

### Go and KEGG enrichment analysis

**Figure 4:**
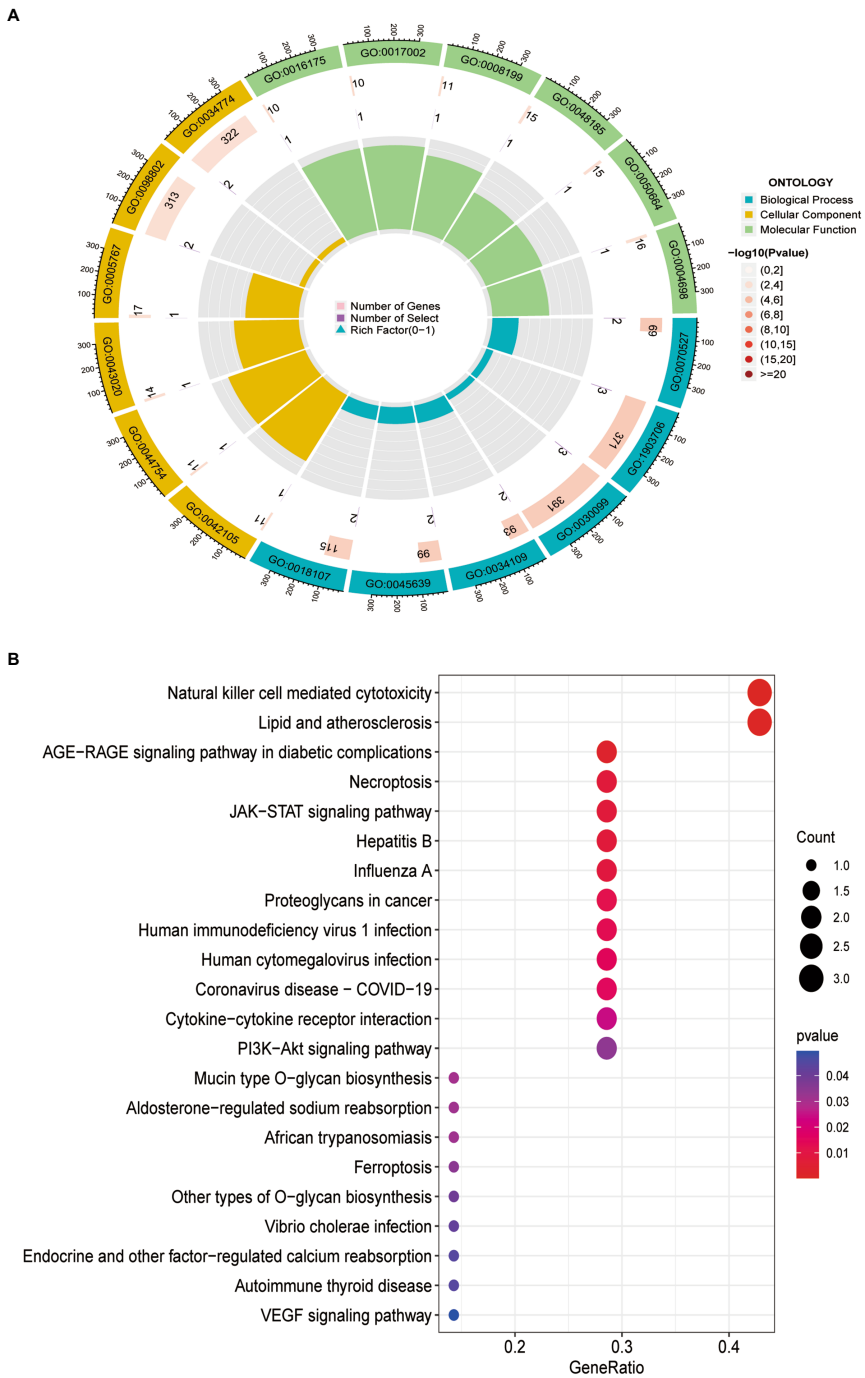
Functional enrichment of 10 DEFRGs. **(A)** GO enrichment analysis. **(B)**. KEGG enrichment analysis.

GO analysis revealed that 10 DEFRGs mainly enriched in platelet aggregation (GO:0070527), regulation of hemopoiesis (GO:1903706), myeloid cell differentiation (GO:0030099), positive regulation of myeloid cell differentiation (GO:0034109) and homotypic cell-cell adhesion (GO:0034109) (Figure 4A). KEGG analysis showed that the pathways enriched by 10 DEFRGs contained natural killer cell-mediated cytotoxicity, lipid and atherosclerosis, and the AGE-RAGE signaling pathway in diabetic complications (Figure 4B).

### Construction and evaluation of LASSO and SVM-RFE models

**Figure 5:**
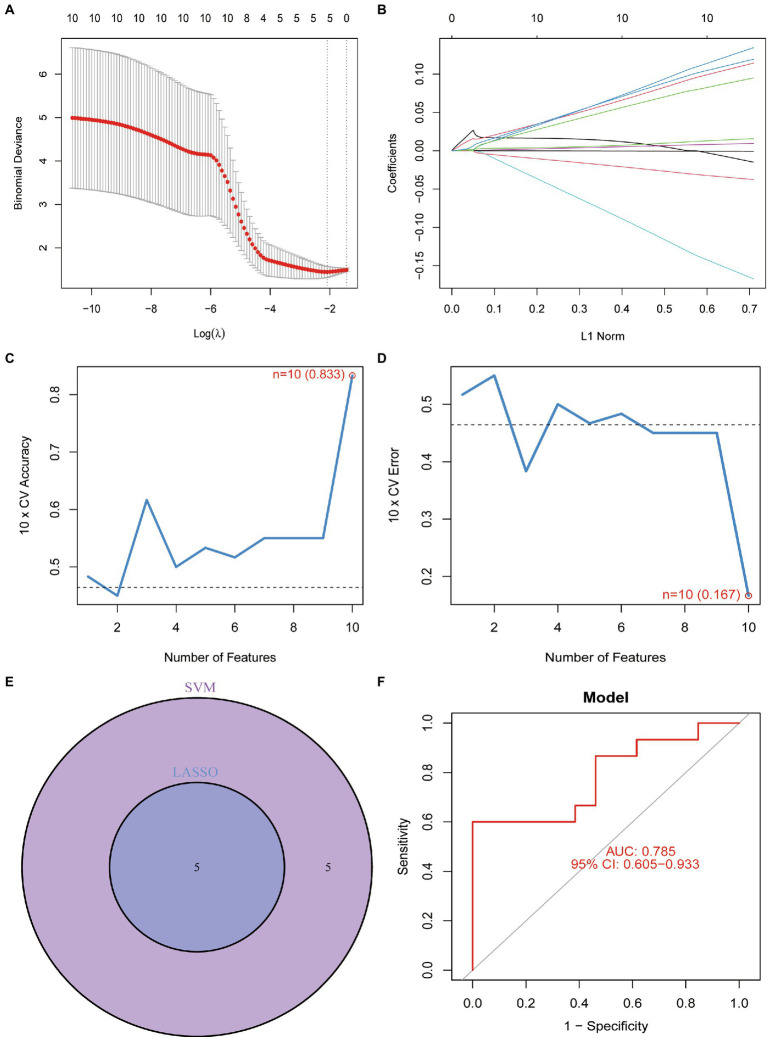
**(A)** Optimal lambda value was selected in the LASSO regression model based on 10-fold cross-validation. **(B)** LASSO coefficient profiles of the five co-expressional DEFRGs. **(C)** Line graph shows the cross-validated accuracy based on different numbers of DEFRGs in the SVM-RFE model. **(D)** Line graph shows the cross-validated error based on different numbers of DEFRGs in the SVM-RFE model. **(E)** Screening of five DEFRGs using LASSO and SVM-RFE algorithms. **(F)** Verification of the diagnostic value of the five DEFRGs by using ROC analysis.

To screen for dependable diagnostic biomarkers related to PCOS, LASSO regression and the SVM-RFE algorithm were performed to evaluate 10 DEFRGs in PCOS. First, the gene expression profiles of the 10 DEFRGs were fit into LASSO regression based on the least squares method. The results revealed that five potential DEFRGs were selected, while the optimal value of lambda was obtained (Figures 5A,B). The SVM-RFE algorithm retained 10 DEFRGs as effective diagnostic biomarkers (Figures 5C,D). Five overlapping DEFRGs (NOX1, ACVR1B, PHF21A, FTL, and GALNT14) were screened as the key DEFRGs for subsequent research (Figure 5E). The AUC value of the overlapping DEFRGs obtained by the SVM-RFE and LASSO regression models was 0.785, indicating accuracy in predicting PCOS (Figure 5F).

### Construction of a nomogram model

**Figure 6:**
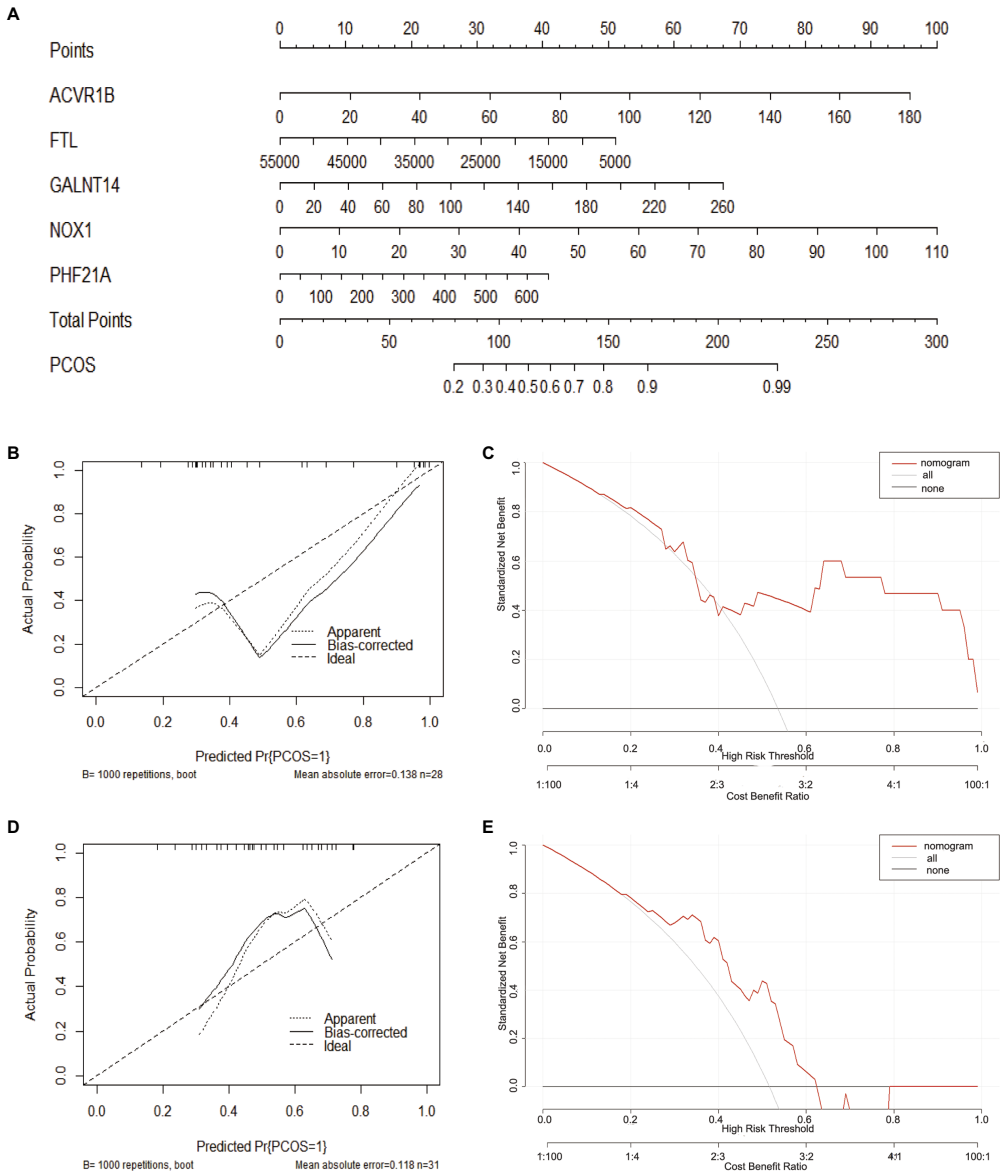
Construction of the nomogram model. **(A)** Construction of the nomogram model based on five key DEFRGs (NOX1, ACVR1B, PHF21A, FTL, and GALNT14). **(B, D)** Calibration curve suggests the accuracy of the nomogram model in the diagnosis of PCOS. **(C, E)** DCA suggests that the nomogram model is of higher clinical application value in the diagnosis of PCOS.

**Figure 7:**
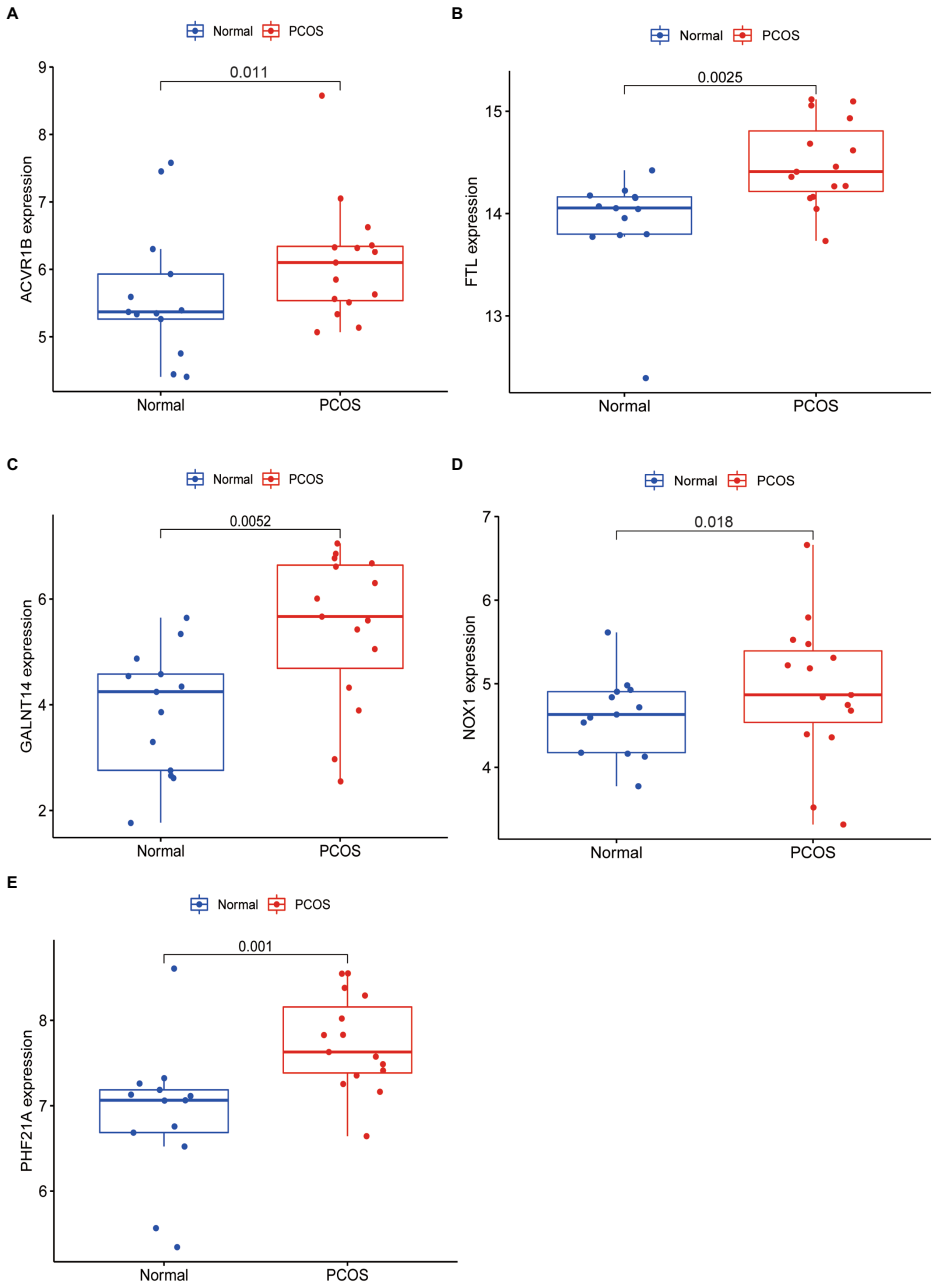
qRT-PCR validated five key DEFRGs differential expression between normal controls and patients with PCOS. **(A)** ACVR1B; **(B)** FTL; **(C)** GALNT14; **(D)** NOX1; and **(E)** PHF21A.

To facilitate the clinical diagnosis of PCOS using selected DEFRGs (NOX1, ACVR1B, PHF21A, FTL, and GALNT14), a nomogram model was constructed (Figure 6A). Calibration curves revealed that the practical diagnostic rate for PCOS based on the nomogram model was close to the ideal diagnostic rate, suggesting the accuracy of the nomogram model for the diagnosis of PCOS (Figure 6B). DCA showed that the net benefits generated by the nomogram model were at the high-risk threshold from 0.1 to 1.0, suggesting that the nomogram model had a higher clinical application value in the diagnosis of PCOS (Figure 6C). We then evaluated the nomogram model based on the RNA expression data of the five selected DEFRGs in our clinical specimens assessed using RT-PCR. Calibration and DCA curves successfully verified the accuracy and net clinical benefit of the nomogram model (Figures 6D,E). In addition, differences in the expressions of NOX1, ACVR1B, PHF21A, FTL, and GALNT14 were verified in our clinical specimens. The results indicated that all five DEFRGs were more highly expressed in patients with PCOS than in normal controls, which is in line with our predictions (Figure 7).

### Gene set enrichment analysis

**Figure 8:**
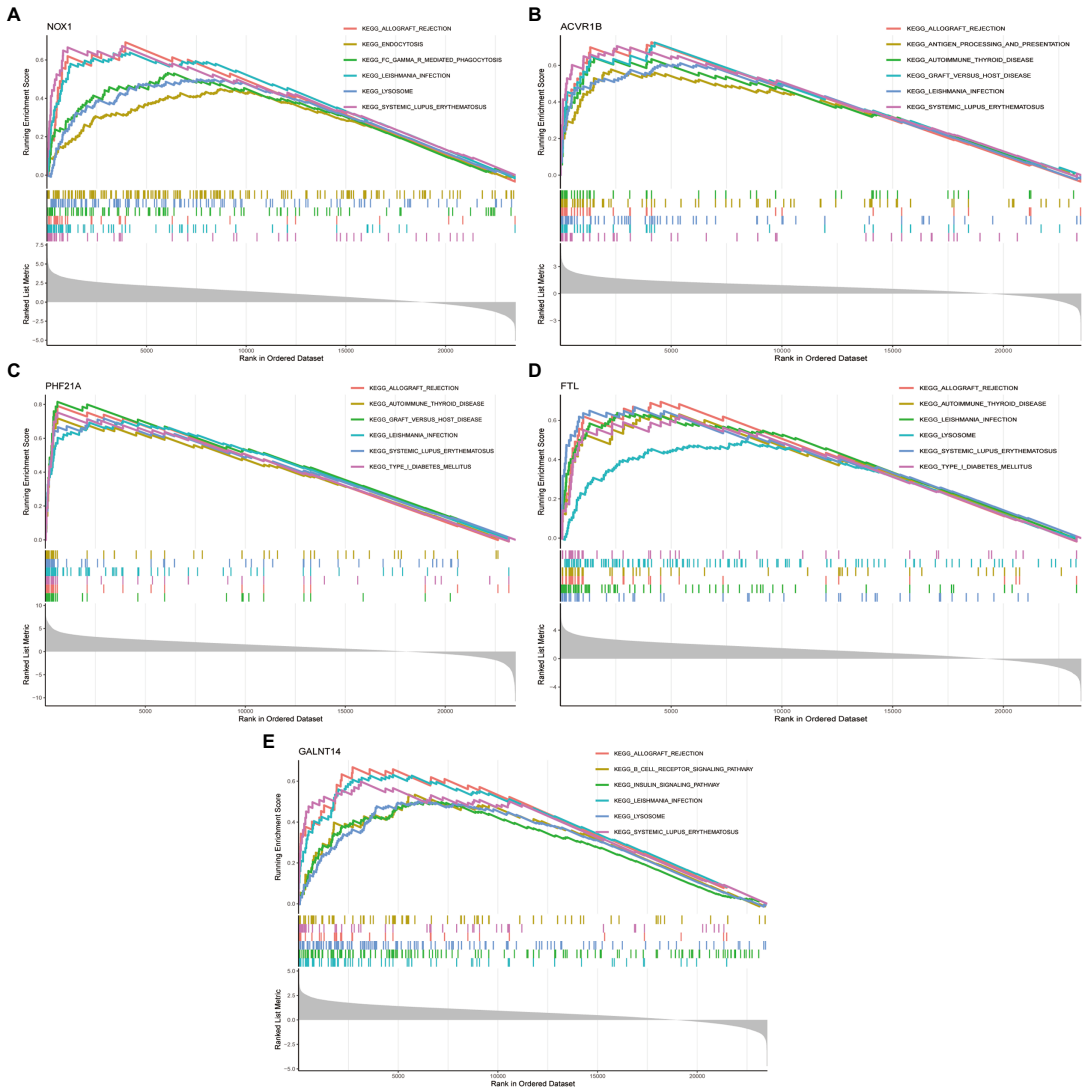
GSEA enrichment analysis showing signaling pathways enriched by five DEFRGs. **(A)** NOX1; **(B)** ACVR1B; **(C)** PHF21A; **(D)** FTL; and **(E)** GALNT14.

We then explored the specific GO function and signaling pathways enriched by the five DEFRGs and the potential mechanisms of the five DEFRGs in the pathogenesis of PCOS. The GSEA results revealed that the main enriched signaling pathways for high NOX1 expression were ALLOGRAFT_REJECTION, ENDOCYTOSIS, FC_GAMMA_R_MEDIATED_PHAGOCYTOSIS, LEISHMANIA_INFECTION, LYSOSOME, and SYSTEMIC_LUPUS_ERYTHEMATOSUS (Figure 8A). The main enriched pathways for high ACVR1B expression were ALLOGRAFT_REJECTION, ANTIGEN_PROCESSING_AND_PRESENTATION, AUTOIMMUNE_THYROID_DISEASE, GRAFT_VERSUS_HOST_DISEASE, LEISHMANIA_INFECTION, and SYSTEMIC_LUPUS_ERYTHEMATOSUS (Figure 8B). The main enriched pathways for high PHF21A expression were ALLOGRAFT_REJECTION, AUTOIMMUNE_THYROID_DISEASE, GRAFT_VERSUS_HOST_DISEASE, LEISHMANIA_INFECTION, SYSTEMIC_LUPUS_ERYTHEMATOSUS, and TYPE_I_DIABETES_MELLITUS (Figure 8C). The main enriched pathways for high FTL expression were ALLOGRAFT_REJECTION, AUTOIMMUNE_THYROID_DISEASE, LEISHMANIA_INFECTION, LYSOSOME, SYSTEMIC_LUPUS_ERYTHEMATOSUS, and TYPE_I_DIABETES_MELLITUS (Figure 8D). Finally, the main enriched pathways for high SNW1 expression were ALLOGRAFT_REJECTION, B_CELL_RECEPTOR_SIGNALING_PATHWAY, INSULIN_SIGNALING_PATHWAY, LEISHMANIA_INFECTION, LYSOSOME, and SYSTEMIC_LUPUS_ERYTHEMATOSUS (Figure 8E).

### Construction of the ceRNA network

**Figure 9:**
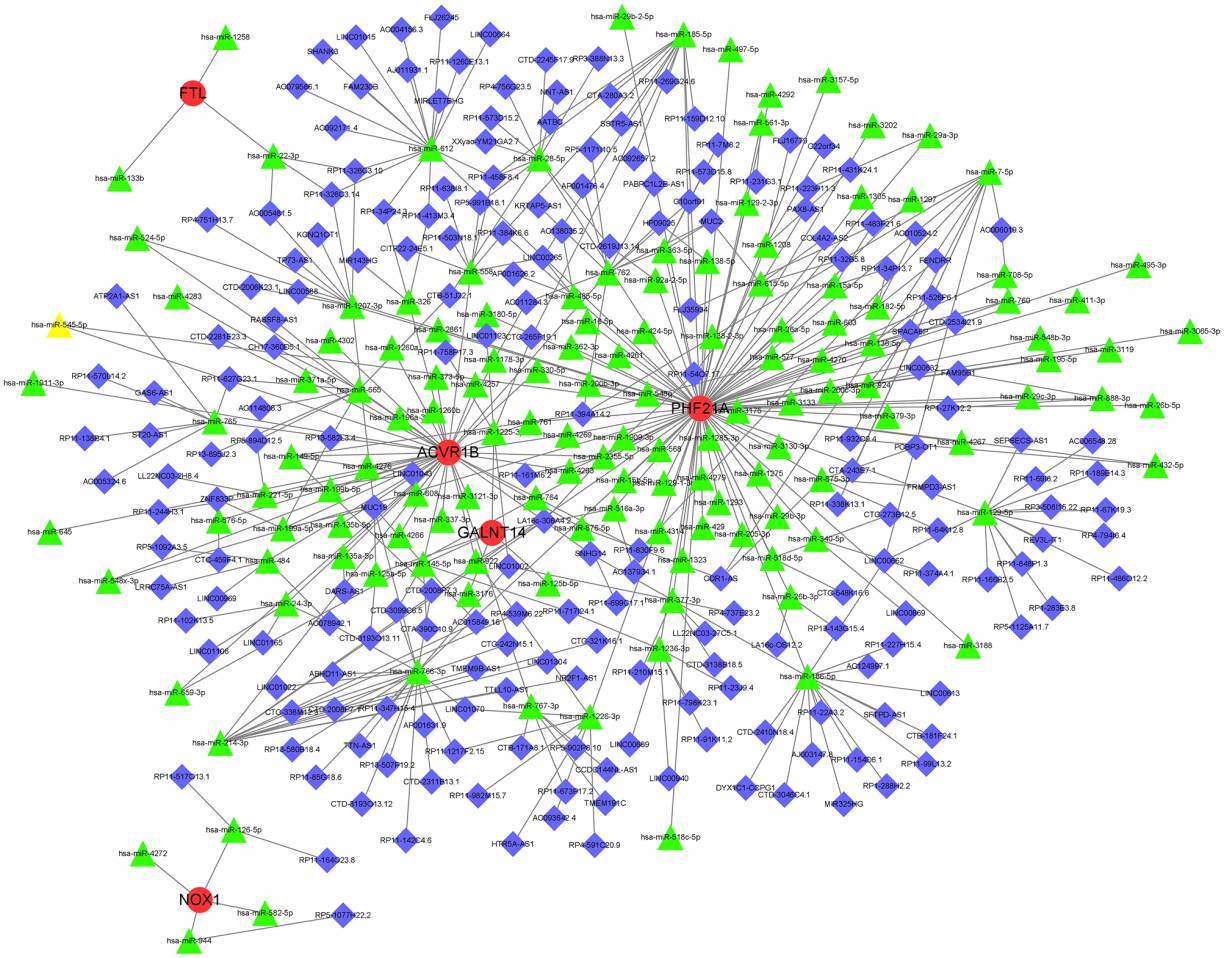
Representative lncRNA–miRNA–mRNA network was obtained by Cytoscape software. Red, green, and blue represent mRNA, miRNA, and lncRNA, respectively.

A lncRNA–miRNA–mRNA network was constructed based on the five key DEFRGs. First, the microRNAs (miRNAs) interacting with the five key DEFRGs were obtained based on the miRWalk database.^4^ The miRNAs that interacted with lncRNAs were acquired from the miRNA target prediction database.^5^ The predicted miRNAs were intersected, and the lncRNA–miRNA–mRNA network included five key DEFRGs, 117 lncRNAs, and 67 miRNAs visualized using Cytoscape software (Figure 9).

## Discussion

In this study, we acquired 10 upregulated DEFRGs based on GEO datasets to explore their roles in the pathogenesis of PCOS using a series of differential expression analyses. GO and KEGG enrichment analyses of 10 DEFRGs indicated that these genes are involved in some pathways associated with immunity and ferroptosis, suggesting that immunity and ferroptosis may be involved in the pathogenesis of PCOS. In addition, five key DEFRGs involved in the diagnosis of PCOS were screened using LASSO regression and the SVM-RFE algorithm. Finally, we constructed a lncRNA–miRNA–mRNA network associated with the five DEFRGs. Increasing evidence indicates that the lncRNA–miRNA–mRNA network plays a critical role in the development of PCOS. For example, Liu G et al. (25) revealed that the lncRNA PVT1/MicroRNA-17-5p/PTEN network was related to the secretion of E2 and P4, proliferation, and apoptosis of granulosa cells in PCOS. Guo et al. (26) illustrated that HOTAIRM1 could sponge miR-433-5p to promote PIK3CD expression, thereby regulating the growth and apoptosis of granulose cells in PCOS. However, the ceRNA network found in our study has not yet been studied. In summary, our study illustrates the potential role of ferroptosis in the pathogenesis of PCOS from the perspective of comprehensive bioinformatics analysis and provides a novel perspective for the clinical diagnosis and treatment of PCOS.

In the present study, five key DEFRGs (NOX1, ACVR1B, PHF21A, FTL, and GALNT14) were identified as the most significant genes related to PCOS pathogenesis, and an accurate and clinically valuable nomogram model was constructed. Ferroptosis is a type of cell death caused by the accumulation of lipid peroxidation products and lethal ROS from iron metabolism (27, 28). The mitochondrial respiratory chain and NADPH oxidase of the NADPH oxidase (NOX) family are major sources of reactive oxygen species (ROS) in human neuronal cells, cardiomyocytes, and keratinocytes (29–31). NOX1, a member of the NOX family, promotes ROS release and ferroptosis (32). Zhang et al. (33) found that ferric ammonium citrate (FAC) increased the ferric content in a human granulosa-like tumor cell line (KGN) by activating the transferrin receptor (TFRC). Iron uptake then mediates the activation of NOX1 signaling, which induces the release of ROS and mitochondrial damage. Therefore, the inhibitory effects of TFRC/NOX1 signaling on follicular genesis may be a potential treatment for PCOS. polypeptide N-acetylgalactosaminyltransferase 14 (GALNT14) is a member of the acetylgalactosaminyltransferase family that can initiate protein O-glycosylation by transferring the GalNAc residue of UDP-GalNAc to the hydroxyl group of Ser or Thr (34). Li et al. (35) indicated that GALNT14 regulates ferroptosis and apoptosis in ovarian cancer by targeting the EGFR/mTOR pathway. Ferritin is the only protein capable of storing substantial amounts of iron, and it plays a key role in regulating cellular iron metabolism. The heavy (H) and light (L) chain subunits of ferritin (FTH and FTL, respectively) are responsible for intracellular iron storage (36). Therefore, FTH1 and FTL levels are positively correlated with ferroptosis and can function as biomarkers of ferroptosis. ACVR1B, also known as ALK-4, acts as a transducer of activin-like ligands that belong to the growth and differentiation factors of the TGF-β superfamily of signaling proteins (37). Kota Fujiki et al. (38) reported that cadmium-and-erabine-induced cell death, including ferroptosis in renal proximal tubular epithelial cells, can be inhibited by blocking the ALK4/5 signaling pathway, suggesting that ALK-4 is related to ferroptosis. PHD finger protein 21A (PHF21A) is also known as BHC80. To date, no relevant studies have reported the mechanism of PHF21A involvement in iron deficiency and PCOS. We selected five key DEFRGs related to PCOS pathogenesis, which will be further elucidated in *in vitro* and *in vivo* experiments.

This study has some limitations. First, our research results were based on bioinformatics analysis. Further basic and clinical experiments are required to verify these results. Second, owing to the limitations of the data in the public database, the sample size included in our study was not large enough, and the research results may deviate from the real situation.

## Conclusion

In the present study, we identified several ferroptosis-related genes that are strongly associated with PCOS pathogenesis, which may provide a novel perspective for the clinical diagnosis and treatment of PCOS. However, further studies are necessary to explore the mechanisms of ferroptosis and its role in PCOS pathogenesis.

## Data availability statement

The datasets presented in this study can be found in online repositories. The names of the repository/repositories and accession number(s) can be found in the article/Supplementary material.

## Ethics statement

The study was approved by the Ethics Committee of the ShengJing Hospital of CMU, and informed consent was obtained from all participants. The patients/participants provided their written informed consent to participate in this study.

## Author contributions

SL, XJ, HG, and FB conceived and designed the study, developed the methodology, analyzed and interpreted the data, wrote, reviewed, and revised the manuscript. All authors contributed to the article and approved the submitted version.

## Funding

This work was supported by 345 Talent Project of Shengjing Hospital of China Medical University (No. M0695); Shenyang Young and Middle-aged Science and Technology Innovation Talents Support Program, Project (No. RC210436); Joint Program of Applied Basic Research of Liaoning Province, Project; Key Laboratory of Reproductive and Genetic Medicine (China Medical University); National Health Commission.

## Conflict of interest

The authors declare that the research was conducted in the absence of any commercial or financial relationships that could be construed as a potential conflict of interest.

## Publisher’s note

All claims expressed in this article are solely those of the authors and do not necessarily represent those of their affiliated organizations, or those of the publisher, the editors and the reviewers. Any product that may be evaluated in this article, or claim that may be made by its manufacturer, is not guaranteed or endorsed by the publisher.

## Footnotes

^1^ http://www.zhounan.org/ferrdb/current/

^2^ http://mirwalk.umm.uni-heidelberg.de/

^3^ http://mirdb.org/

^4^ http://mirwalk.umm.uni-heidelberg.de/

^5^ https://miRDB.org
