# Supplementary material for: Relationships of ferroptosis-related genes with the pathogenesis in polycystic ovary syndrome
Source: Front Med (Lausanne). 2023 Feb 17;10:1120693. doi: 10.3389/fmed.2023.1120693 (PMC9981782; doi:10.3389/fmed.2023.1120693)
Supplement: Supplementary file 1 [file Table_1.docx]

Supplementary table 1: Primer sequences of the 5 key DEFRGs for reverse transcription- quantitative polymerase chain reaction.

| **Gene** | **Forward (5′-3′)** | Reverse (5′-3′) |
| --- | --- | --- |
| GALNT14 | 5'-TTGATGAGCGGCGGTATCTGAATG-3' | 5'-TGCGGTTTAATACACTGCGGATGG-3' |
| FTL | 5'-GCGATGATGTGGCTCTGGAAGG-3'， | 5'-TGTGGAGGTTGGTCAGGTGGTC-3'; |
| PHF21A | 5'-GTTTCTCTCCTGCTGCTCTGATTCC-3' | 5'-CCTCCTCCTCCTCCTCCTCTCC-3' |
| ACVR1B | 5'-CCCTGCTGACCTCCCACCTATC-3' | 5'-GCTTCCCAAAGACCTGCTCCATC-3' |
| NOX1 | 5'-TGCCTCCATTCTCTCCAGCCTATC-3' | 5'-AATGCCGTGAATCCCTAAGCCAAG-3' |
| GAPDH | 5'-TGACAACTTTGGTATCGTGGAAGG-3' | 5'-AGGCAGGGATGATGTTCTGGAGAG-3' |
